# Epilepsy as a consequence of infantile vitamin B12 deficiency: observations from a prospective cohort study and a review of the literature

**DOI:** 10.3389/fnut.2026.1903748

**Published:** 2026-10-08

**Authors:** Pawan Kumar, Sujatha Manjunathan, Sameer Vyas, Jitendra Kumar Sahu, Naveen Sankhyan

**Affiliations:** 1 Department of Pediatric Neurology, Dr. Yashwant Singh Parmar Government Medical College & Hospital, Nahan, Sirmaur, Himachal Pradesh, India; 2 Department of Pediatrics, Postgraduate Institute of Medical Education and Research, Chandigarh, India; 3 Department of Radiodiagnosis and Imaging, Postgraduate Institute of Medical Education and Research, Chandigarh, India

**Keywords:** cobalamin deficiency, infantile epileptic spasms syndrome (IESS), infantile tremor syndrome, infantile vitamin B12 deficiency, infantile-onset epilepsy, ITS, Neurocutaneous Infantile B12 deficiency Syndrome

## Abstract

**Introduction:**

Symptomatic infantile vitamin B12 deficiency has far-reaching neurological consequences for affected children. Development of epilepsy is one such consequence whose incidence and characteristics have been scarcely studied and reported in the literature.

**Objective:**

To describe the incidence, characteristics, and outcomes of epilepsy in a prospective cohort of children treated for infantile vitamin B12 deficiency and to briefly review the published literature on the topic.

**Methods:**

A hospital-based prospective cohort study enrolling consecutive children with symptomatic infantile vitamin B12 deficiency was conducted from January 2021 to June 2023 to study the neurological consequences of deficiency. The cohort was followed through March 2026 to determine the incidence of epilepsy during follow-up. Those who developed epilepsy were evaluated for seizure and epilepsy types, neuroimaging and electroencephalography findings, treatment, response to treatment, and outcomes. Multivariable Cox proportional hazards modeling was performed using baseline predictors identified through univariate screening to determine independent associations with epilepsy.

**Results:**

A total of 141 children with infantile vitamin B12 deficiency were part of the original cohort. Four died, 25 were lost to follow-up. Among the rest of the 112 children, at a median follow-up of 51 months (IQR: 45–55), 31 (28, 95% CI 19–36%) developed epilepsy. In those with epilepsy, the mean age at diagnosis of infantile vitamin B12 deficiency was 12.39 ± 3.9 months, and epilepsy developed after a mean interval of 4.7 ± 3.2 months after treatment. Epileptic spasms were the most common seizure type (15/31, 48.4%), while other forms of epilepsy were observed in 16. Of the 31, at last contact, 21 (68%) had either resolved or well-controlled epilepsy, 8 (26%) had drug-refractory epilepsy, and 2 (6%) died. In the multivariate model, acute-phase seizures were strongly associated with increased epilepsy risk (pooled HR 4.27, 95% CI:1.6–11.5, *p* = 0.004). Higher follow-up DQ was protective (pooled HR 0.94, 95% CI:0.89–0.99, *p* = 0.015). There was an early clustering of epilepsy, with all events occurring within 10 months of follow-up.

**Conclusion:**

Epilepsy is a significant neurological outcome of vitamin B12 deficiency in infants. There is early clustering of epilepsy risk after treatment for deficiency, and acute-phase seizures are the strongest independent predictor of later epilepsy.

## Introduction

Infantile vitamin B12 deficiency is commonly encountered in predominantly vegetarian regions of the Indian subcontinent (1). The deficiency typically affects an exclusively or predominantly breastfed child of a vegetarian mother. The typical cutaneous and hair manifestations, pallor, with developmental delay or regression and variable presence of movement disorders, helps in clinical identification of this syndrome, also called the Neurocutaneous Infantile B12 deficiency Syndrome (NIBS) (1, 2). There has been a growing body of evidence suggesting the long-term neurodevelopmental consequences of B12 deficiency in infancy despite treatment and normalization of blood levels of B12 and homocysteine (3–5). The commonly encountered consequences are poor head growth leading to microcephaly, developmental delays of variable degrees, autistic traits, epilepsy, and, rarely, death (1, 3–5). The long-term presence of these neurodevelopmental morbidities in children with B12 deficiency underscores the critical role of vitamin B12 in brain growth, maturation, and developmental progression (6).

Epilepsy, if seen as a sequel, may add to the developmental morbidities seen in these children. The description of epilepsy in children treated for infantile B12 deficiency is limited to case reports, case series, and small retrospective observational studies (7–9). Hence, we aimed to determine the incidence of epilepsy by following up a previously published prospective cohort of children with symptomatic vitamin B12 deficiency. The objectives were to evaluate the seizure and epilepsy types, neuroimaging and electroencephalography findings, and response to treatment in children who developed epilepsy.

## Materials and methods

This hospital-based cohort study was a follow-up of the prospective cohort study, conducted from January 2021 to June 2023 at a tertiary care hospital for children in India, aimed at studying the neurological consequences of Infantile vitamin B12 deficiency (3). The study was approved by the Institute’s ethics committee (Ref.no. INT/IEC/2021/SPL-707, INT/IEC/2022/SPL-127, IEC-INT/2026/Study-4159). There were 141 children enrolled in the study. This cohort consisted of consecutive children with proven Infantile vitamin B12 deficiency. All children in the parent study (3) had a baseline assessment of clinical symptoms and signs, including anthropometric indices, skin and hair changes, and neurological findings. Also, a baseline development assessment using CAPUTE scales: Cognitive Adaptive Test (CAT)/ Clinical Linguistic & Auditory Milestone (CLAMS) was available for all children. Baseline hemogram (measured by an automated cell Counter - HmX500, Beckman Coulter, USA/XN-1000, Sysmex, Japan), serum vitamin B12 levels (measured by competitive immunoassay using ADVIA Centaur and ADVIA Centaur XP systems), and plasma homocysteine levels (measured by competitive immunoassay using ADVIA Centaur system) were available in all children. The lower normal limit of serum vitamin B12 was 211 pg/ml, and the higher normal limit of plasma homocysteine levels was 15 mmol/L. Data on baseline Magnetic Resonance Imaging (MRI) of the brain, consisting of the following sequences: 3D T1-weighted axial spoiled gradient recalled, T2-weighted axial, Fluid Attenuated Inversion Recovery (FLAIR) axial, diffusion weighted imaging (DWI) sequences, done in a 3.0 Tesla MR Scanner using a standard 32-channel head coil (Ingenia, Philips Healthcare, Netherlands), was available for all children, and a follow-up imaging done after 3 months or more after the baseline MRI.

All surviving children were followed up till March 2026. During the follow-up period, children who developed epilepsy were evaluated. The diagnosis of epilepsy was made in those who fulfilled the 2014 International League Against Epilepsy criteria for diagnosis of Epilepsy [(i) at least two unprovoked (or reflex) seizures that occur more than 24 h apart; (ii) one unprovoked (or reflex) seizure and a probability of additional seizures that is comparable to the general recurrence risk (at least 60%) following two unprovoked seizures, occurring over the next 10 years; and (iii) an epilepsy syndrome diagnosis] (10). Age at onset of epilepsy, epilepsy type/syndrome were recorded. At least one electroencephalogram was performed for 30 min or more on all children who developed epilepsy in follow-up. Records of the electroencephalogram, therapy details, and response to treatment were reviewed. Those who were seizure-free for at least 12 months with or without anti-seizure medications were labeled as having controlled epilepsy. Those with seizure recurrence despite treatment with two or more anti-seizure medications, excluding hormonal therapy, were labeled as drug-refractory. The collected data were entered into the Microsoft Access database.

Statistical analyses were performed using IBM SPSS Statistics for Windows, version 29.0.1.0(171) (IBM Corporation, Armonk, New York, USA). Descriptive data were expressed as numbers and proportions. Numerical data were expressed as Mean ± Standard Deviation (SD) or Median and Interquartile ranges (IQR). Paired t-test was employed to analyze paired dependent quantitative variables, unpaired t-test was used to analyze independent quantitative variables, and the chi-square test or Fisher’s Exact test was used to analyze two or more qualitative variables. A *p*-value of less than 0.05 was considered significant. Missing follow-up developmental quotient (DQ) values were addressed using Multiple Imputation with 20 fully conditional specifications using predictive mean matching. Time-to-epilepsy was evaluated using Kaplan–Meier curves and multivariable Cox proportional hazards regression, with variables with univariate *p* ≤ 0.20 considered for model entry. Analyses were pooled using Rubin’s rules, and proportional hazards assumptions were confirmed through log–minus–log plots and time-dependent covariates. Time-to-event analysis was conducted to evaluate cumulative epilepsy-free survival across stratified patient cohorts. The primary outcome was the time to an epileptic event, with patients remaining event-free at the conclusion of observation being right-censored at their last recorded follow-up date. Non-parametric Kaplan–Meier curves were constructed to estimate epilepsy-free survival across the group stratifications that emerged after the multivariable Cox proportional hazards regression, namely, Acute-phase seizures present or not, and groups dichotomized based on the median of the observed DQ.

For the literature review, a standard search strategy was adopted. A thorough search of electronic databases, including PubMed and Embase, was performed for studies on epilepsy in Infantile vitamin B12 deficiency. The MeSH terms used for the search strategy were Infantile vitamin B12 deficiency [OR] Infantile tremor syndrome [OR] Vitamin B12 deficiency in infancy [OR] Vitamin B12 deficiency in children [AND] epilepsy [OR] seizures [OR] epileptic spasms [OR] spasms [OR] West syndrome [OR] Infantile Epileptic Spasms Syndrome [OR] IESS. The search returned a total of 757 articles, including case reports, case series, and observational studies. After removing duplicates, articles were screened to exclude the following: (i) published abstracts presented in conferences, (ii) non-English articles, (iii) articles that reported seizures or epileptic spasms at the time of diagnosis of infantile vitamin B12 deficiency and not during follow-up, (iv) those that reported characteristics of epilepsy associated with inherited cobalamin pathway defects, and other genetic causes related to vitamin B12 absorption and metabolism, and (v) unrelated articles. After exclusion, eight articles reporting on the development of epilepsy and/or outcomes in children treated for infantile vitamin B12 deficiency were included in the final review (Supplementary Figure 2). Supplementary Table 1 summarizes available information on the onset and characteristics of epilepsy in follow-up of treated children from previous studies.

## Results

The cohort of 141 children who were treated for Infantile B12 deficiency was followed through March 2026 to assess epilepsy incidence and outcomes. Four died during the initial phase of follow-up, and 25 were lost to follow-up. Of the remaining cohort of 112 children, two died due to epilepsy. The remaining 110 children had a median follow-up of 51 months (IQR: 45–55), with similar medians in those with epilepsy [50 (IQR: 45–54)] and those without epilepsy [52 (IQR: 45–56)]. Thirty-one children [28%; (95%CI: 19–36%)] developed epilepsy, of whom two had died due to epilepsy-related complications (Supplementary Figure 1).

The baseline characteristics, including age, gender, growth indices, pertinent clinical features, serum B12 and plasma homocysteine levels, brain MRI findings at the time of B12 deficiency diagnosis, and baseline neurodevelopmental assessment, are summarized in Table 1. In univariate analysis, factors significantly associated with epilepsy occurrence were the presence of seizures at diagnosis and a basal ganglia signal change on MRI at diagnosis (Supplementary Table 2). The children with a diagnosis of epilepsy had a significantly lower mean developmental quotient at follow-up, even prior to the onset of epilepsy.

**Table 1:** Summary of characteristics of children with infantile B12 deficiency at diagnosis who developed epilepsy on follow-up (*N* = 31).

Parameter	Observation
Mean age at diagnosis of Infantile B12 deficiency	14(11–18)
Boys	19
Presenting clinical features	
Developmental delay	29/31 (93.5%)
Milestone regression	20/31 (64.5%)
Pallor	31/31 (100.0%)
Lethargy	29(93.5%)
Skin hyperpigmentation	28/31 (90.3%)
Hair changes	30/31 (96.8%)
Nail changes (leukonychia/platynychia)	7/31 (22.6%)
Weak cry	25/31 (80.6%)
Tremors	24/31 (77.4%)
Seizures	5/31 (16%)
Growth indices at diagnosis	
Weight for age (*Z*-score)	−1.48 ± 1.4
Length for age (*Z*-score)	−1.96 ± 1.13
Weight for length (*Z*-score)	−0.40 ± 1.78
Head circumference for age (*Z*-score)	−2.03 ± 1.16
Head circumference for age less than −2 *Z*-score (Small head)	17/31 (54.8%)
Head circumference for age less than −3 *Z*-score (Microcephaly)	7/31 (22.6%)
Exclusively breastfed	28/31 (90.3%)
Biochemical profile	
Hemoglobin	8.2 ± 1.8 g/dL
Serum B12 levels	422.7 ± 539.4 pg./mL
Low serum vitamin B12 (less than 211 pg./ml)	17/31 (54.8%)
Mean plasma homocysteine levels	47.2 ± 27.1 μmol/L
High homocysteine (more than 15 mmol/L)	30/30 available (100%)
MRI at diagnosis	
Normal	1
Frontotemporal atrophy	29 (93.5%)
White matter atrophy	25(81%)
Corpus Callosum Atrophy	26(84%)
Basal Ganglia Signal changes	5(16%)
Cerebellar atrophy	23(74%)
MRI at follow-up*	
Frontotemporal atrophy	10(32%)
White matter atrophy	4(13%)
Corpus Callosum Atrophy	13(42%)
Basal Ganglia Signal changes	2(6.5%)
Cerebellar atrophy	6(19%)
Developmental assessment	
Baseline full-scale DQ	19.8 ± 12.3
Follow up full-scale DQ	44 ± 9

* Done median 120 days (IQR 98–200) after the first MRI.

The mean age at the onset of epilepsy was 19.0 ± 7.1 months. The mean interval to epilepsy onset from diagnosis of Infantile B12 deficiency was 4.7 ± 3.2 months. Generalized seizures were seen in 24 of the 31 children (77.4%) and focal seizures in 7 children (22.6%). The most common generalized seizure type was epileptic spasms, seen in 15 children (48.4%) who developed epilepsy. Generalized clonic seizures were seen in 6 children (19.4%), and generalized tonic–clonic seizures were seen in 3 children (9.7%). All the 7 children with focal seizures had focal clonic type of seizures. A single type of seizure was seen in all 31 children at the onset of epilepsy (Table 2).

**Table 2:** Summary of epilepsy characteristics and outcomes in children treated for Infantile B12 deficiency.

**Parameter**	**Observation**
Mean age at onset of epilepsy (N = 31)	19.9 ± 7.1 months
Mean interval to epilepsy onset from diagnosis of B12 deficiency	4.7 ± 3.2 months
Median follow-up after epilepsy onset	28 months (IQR: 5–60 months)
ILAE-based type of seizure and epilepsy classification: (*N* = 31)	*N* (%)
*Generalized seizures/epilepsy*	24 (77.4%)
Epileptic spasms & IESS	15 (48.4%)
Generalized clonic seizures	6 (19.4%)
Generalized tonic–clonic seizures	3 (9.6%)
*Focal clonic seizures/epilepsy with impaired consciousness*	7 (22.6%)
ASM requirement: (*N* = 31)	
1 ASM	9 (29%)
2 ASMs	10 (32.3%)
3 ASMs	12 (38.7%)
Outcomes	
Overall mortality	2 (6.5%)
Electroclinical resolution of spasms in children with IESS (*N* = 15)	15 (100%)
Proportion of children off-ASMs in last follow-up (*N* = 29)	9 (31%)
Proportion of children on 1 or more ASMs in last follow-up (*N* = 29)	20 (69%)

All 15 (48.4%) with IESS had hypsarrhythmia as the interictal finding, with abnormal background and poor sleep architecture for age. Generalized interictal epileptiform discharges (IEDs) were seen in 9 children (29%), and focal IEDs were seen in 7 children (22.6%) (Table 3).

**Table 3:** Interictal EEG findings in children treated for Infantile B12 deficiency developing Epilepsy in follow-up (*N* = 31).

**Epilepsy type**	***n***	**Principal EEG pattern**	**Description of IEDs and background**
Infantile Epileptic Spasms Syndrome (IESS)	15	Hypsarrhythmia, 15/15 (100%)	IEDs: Multifocal spikes, polyspikes, and spike/polyspike-wave epileptiform discharges, frequent to abundant, occurring in irregular bursts or clusters, followed by prominent slowing in 15/15 (100%) Background: High-amplitude disorganized slow background with poor age-appropriate sleep architecture in 15/15 (100%)
Generalized epilepsy	9	Bilaterally synchronous generalized IEDs, 9/9 (100%)	IEDs: Predominantly generalized spike-and-wave, sharp or spike-and-slow-wave discharges, with bilateral synchrony and an anterior predominance. The distribution of dominance was bilateral frontotemporal in 4/9 (44.4%), frontocentral in 3/9 (33.3%), and frontoparietal in 2/9 (22.2%). Background: Age-appropriate sleep background in 9/9 (100%)
Focal epilepsy	7	Focal/regional IEDs, 7/7 (100%)	IEDs: Predominantly focal spikes, sharps, or spike and wave complexes Regional localization was frontotemporal in 4/7 (57.1%), frontocentral in 2/7 (28.6%), and frontoparietal in 1/7 (14.3%). IEDs were left-sided in 4/7 (57.1%) and right-sided in 3/7 (42.9%). Background: Age-appropriate sleep background in 7/7 (100%)

The brain MRI done at the time of diagnosis showed abnormalities in 30 (96.8%) of the children. A follow-up brain MRI was performed in 30 children with abnormalities on baseline imaging, after a median interval of 120 days from the first MRI, showing residual abnormalities in a sizeable proportion (Table 1).

The 15 children with IESS were treated with intramuscular injections of Adrenocorticotropic hormone (ACTH), and one out of the 15 with IESS required the addition of vigabatrin in view of persistent epileptic spasms despite ACTH therapy. All of those with IESS had a documented electro-clinical resolution of epileptic spasms after therapy. Of those with IESS, 4 did not require any other therapy apart from ACTH and remain seizure-free. Among the rest, 5 developed drug-refractory epilepsy with other seizure types (Supplementary Table 3). Among those with generalized epilepsy, two of the nine developed drug-refractory epilepsy, while one of the seven with focal epilepsy developed drug-refractory epilepsy (Supplementary Figure 1). Overall, 31 [28%; (95% CI: 19–36%)] of 112 children developed epilepsy, 21 (68%) had either resolved or well-controlled epilepsy, 8 (26%) had drug-refractory epilepsy, and 2 (6%) died.

In the multivariable Cox proportional hazards model, acute-phase seizures were strongly associated with increased epilepsy risk (pooled HR 4.27, 95% CI: 1.6–11.5, *p* = 0.004), indicating that children with seizures at the time of diagnosis had more than a fourfold higher hazard of developing epilepsy compared with those without seizures. Higher follow-up developmental quotient (DQ) was independently protective (pooled HR 0.94, 95% CI: 0.89–0.99, *p* = 0.015), with each 1-point increase in DQ reducing epilepsy hazard by approximately 6%. MRI signal abnormalities, cortical atrophy, sex, socioeconomic status, tremors, and age were not significant predictors in the imputed model and were excluded from the final adjusted model. The proportional hazards assumption was satisfied for both retained predictors, supported by parallel log–minus–log curves and non-significant time-interaction terms. Kaplan–Meier analysis showed significantly lower epilepsy-free survival in children with acute-phase seizures and in those with a developmental quotient below the median value of 46, with both comparisons demonstrating clear early separation of survival curves (log-rank *p* < 0.001 for seizures; *p* = 0.021 for DQ). These patterns were consistent with the multivariable Cox findings, where seizures and follow-up DQ remained the only independent predictors of epilepsy (Figure 1). The Kaplan–Meier curves also demonstrated clear early clustering of epilepsy events within the first 10 months after diagnosis.

**Figure 1:**
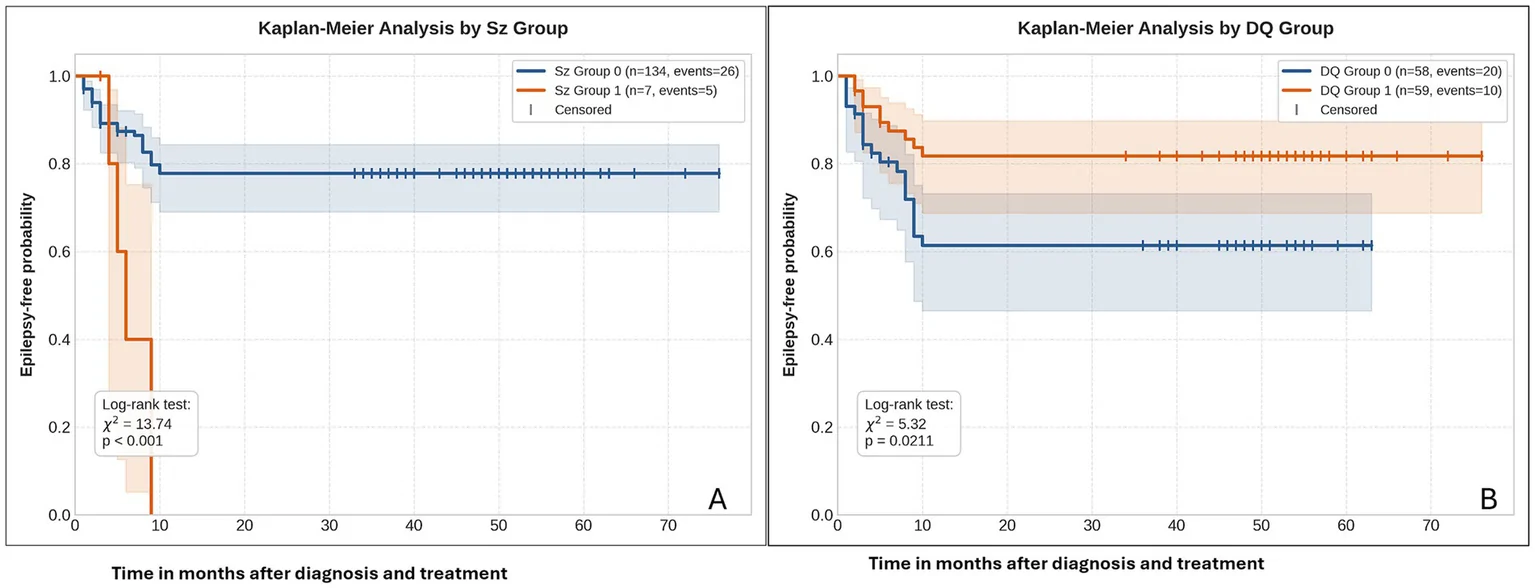
Kaplan–Meier epilepsy-free survival curves stratified by acute-phase seizures (Sz) **(A)** and follow-up developmental quotient categories **(B)**. **A** shows a steep early decline in epilepsy-free survival among infants with acute-phase seizures, whereas those without seizures maintained high epilepsy-free survival throughout follow-up. **B** demonstrates higher epilepsy-free survival among children with follow-up developmental quotient (DQ) values below the median (46) compared with the rest. The curves demonstrated clear early clustering of epilepsy events within the first 10 months. Censored observations are indicated by tick marks.

## Discussion

This study shows that epilepsy is an important long-term consequence of symptomatic infantile vitamin B12 deficiency. Among those who survived the initial illness, 28% later developed epilepsy, and two died from epilepsy-related complications. Acute-phase seizures were the most powerful predictor of subsequent epilepsy, conferring more than a fourfold increase in risk. The Kaplan–Meier curves demonstrated clear early clustering of epilepsy events within the first 10 months, indicating that the period immediately after diagnosis and treatment represents the highest-risk window for epileptogenesis. Similarly, children with higher DQ values showed consistently better epilepsy-free survival than those with lower DQ, possibly indirectly indicating the relationship of severity and duration of deficiency with long-term epilepsy risk.

The median age of onset of epilepsy, with only Epileptic spasms being the only reported type of epilepsy in the available literature in the previously reported patients, was 10.5 months, around 3 months after the diagnosis of vitamin B12 deficiency. In our cohort, epilepsy began at a mean age of 19.9 months, approximately 4.7 months after the diagnosis of B12 deficiency. The mean age of onset of epilepsy was later in our cohort compared to 6.5 months in the largest retrospective cohort by Sharawat et al. (9). The major caveat in the interpretation of this slightly later age at onset of epilepsy in our cohort with the previously published patients is that the previously available patient data are mostly case reports or series, which are subject to reporting and publication bias. This timing suggests that epilepsy may be a delayed consequence of metabolic brain injury caused by early deficiency. Only 5 of the 31 children had seizures during the acute, untreated phase. The interval between nutritional correction and seizure onset suggests that early B12 deficiency triggers neurodevelopmental vulnerabilities such as delayed myelination, impaired synaptic maturation, and increased cortical excitability that become clinically apparent only months later (11, 12). This interpretation is consistent with neuroimaging and neurophysiological evidence showing that the effects of early cobalamin deficiency can persist well beyond hematologic recovery (3). The mechanisms underlying epilepsy and epileptic spasms in these children remain uncertain because this subgroup has been poorly studied. In rat models, pretreatment with vitamin B12 reduces seizure frequency in PTZ-kindled animals, suggesting a possible antiepileptogenic effect of adequate B12 supplementation (13). Evidence from animal studies and adult reports also suggests that B12 deficiency-related myelin dysfunction may enhance glutamate signaling, the brain’s main excitatory neurotransmitter, thereby promoting epileptogenesis (11, 12). Chronic methylcobalamin use has also been shown to protect cortical neurons from NMDA receptor-mediated glutamate toxicity (14). Taken together, these observations support the possibility that vitamin B12 deficiency increases susceptibility to epilepsy in children. Seizure presentation in this cohort was predominantly generalized: 77% of affected children had generalized seizures, and nearly half presented with epileptic spasms or infantile epileptic spasms syndrome (IESS). This prominence of spasms is notable because IESS is usually linked to structural, metabolic, or genetic causes. Early B12 deficiency may act as a global encephalopathic insult, predisposing children to epileptic spasms in a way similar to other early brain injuries. Encouragingly, all 15 children with spasms achieved electroclinical resolution, suggesting that despite presentation with spasms, this phenotype may respond better to treatment than spasms caused by classic structural etiologies. Current evidence on epilepsy after treatment of infantile B12 deficiency is limited largely to case reports, small case series, and retrospective studies (7, 15–19) (Supplementary Table 1). In our cohort, epileptic spasms were the most common form of epilepsy, similar to patterns seen after other early brain insults and in keeping with the scarce literature on the subject reviewed by us (Supplementary Table 1). In addition, lower vitamin B12 levels have been observed in children with epileptic spasms compared with children who have developmental delay without spasms, suggesting a possible link (20). In univariate analysis, epilepsy was significantly associated with seizures at diagnosis and basal ganglia signal changes on MRI at diagnosis (Supplementary Table 2). From a clinical standpoint, seizures during the acute phase are particularly important because affected children may remain at risk of developing epilepsy even after apparent recovery from the vitamin deficiency, warranting closer follow-up in these children. Notably, children who later developed epilepsy already had significantly lower developmental quotients at follow-up before epilepsy onset. This, along with the involvement of the basal ganglia, which indicates a final energy failure in severe vitamin B12 deficiency, suggests that epilepsy may be a marker of the severity of the deficiency. The presence of drug-refractory epilepsy and epilepsy-related deaths underscores the severity of this condition and highlights the importance of early diagnosis, timely nutritional rehabilitation, and close neurological follow-up.

The findings of this study have important clinical implications. Firstly, children treated for infantile vitamin B12 deficiency require ongoing neurological monitoring, particularly during the first few months after treatment, because epilepsy may develop after a delay. Secondly, the high rate of epileptic spasms suggests that clinicians should have a low threshold for performing an EEG in children with developmental regression or new-onset seizures following B12 deficiency. Those who had seizures at the time of diagnosis appear to be at risk for later epilepsy. In addition, although seizure control is often possible in most, the presence of drug-refractory epilepsy and epilepsy-related deaths underscores the severity of this condition. Finally, despite all care, developmental outcomes remain a concern, underscoring the need for early developmental support and long-term neurocognitive follow-up.

Acute-phase seizures were the most powerful predictor of subsequent epilepsy, conferring more than a fourfold increase in risk. This strong association reinforces the concept that early seizures reflect greater underlying brain vulnerability and a heightened propensity for epileptogenesis, as well as a longer period of deficiency impairing protective mechanisms. Conversely, a higher follow-up DQ was independently protective, with each incremental improvement reducing epilepsy hazard. The lack of significant associations with MRI signal abnormalities, cortical atrophy, tremors, or age indicates that, within this population, acute-phase seizures and developmental status exert a dominant influence over structural or demographic factors. The early and persistent separation of Kaplan–Meier curves complements the adjusted hazard estimates and underscores the clustering of epilepsy events among clinically vulnerable subgroups.

Several limitations should be considered when interpreting the findings of this study. Although the cohort initially included 141 children, only 112 had evaluable outcomes. With only 31 children developing the outcome of interest, the study has limited statistical power to estimate incidence with high precision; the resulting confidence intervals are wide, and small shifts in the number of events could meaningfully alter the incidence estimate. This also limited the number of covariates that could be included in the Cox model and may reduce the stability of estimates for less common predictors. The outcomes of the children lost to follow-up remain unknown, and even a modest number of additional events among them could substantially change the observed incidence and its interpretation. This may have influenced time-to-event estimates despite censoring. Multiple imputation was used to address missing developmental data, but imputed values may not fully capture the true variability of neurodevelopmental trajectories. Secondly, some associations we observed may be chance findings, particularly given the multiple comparisons performed. Third, epilepsy in early childhood is dynamic: diagnoses may evolve, seizure patterns may change, and treatment responses may vary over time. Children who were seizure-free at assessment may later develop epilepsy, whereas those in remission may relapse. These changes could influence outcome patterns and limit the stability of current inferences. Overall, these limitations highlight the need for a well-designed, adequately powered cohort with systematic long-term follow-up and minimal attrition to define more clearly the course of epilepsy and neurodevelopmental outcomes after infantile vitamin B12 deficiency.

In summary, epilepsy developing after infantile vitamin B12 deficiency appears to be an underrecognized and potentially preventable cause of childhood epilepsy. This is especially important in regions where nutritional deficiency remains common. From a public health perspective, prevention of infantile B12 deficiency through maternal supplementation, dietary counseling, and early screening of high-risk groups may be the most effective way to reduce the long-term neurological sequelae observed in this cohort.

## Data Availability

Data can be made available on request in line with institutional policies and applicable ethical and legal requirements. Requests will be reviewed and, where approved, are subject to a signed Data Sharing Agreement prior to release.
