# Supplementary material for: Epilepsy as a consequence of infantile vitamin B12 deficiency: observations from a prospective cohort study and a review of the literature
Source: Front Nutr. 2026 Oct 8;13:1903748. doi: 10.3389/fnut.2026.1903748 (PMC13687030; doi:10.3389/fnut.2026.1903748)
Supplement: Supplementary file 1 [file Supplementary_file_1.pdf]

Supplemental Figure 1: Flow diagram of the patient cohort from initial enrolment to epilepsy outcomes

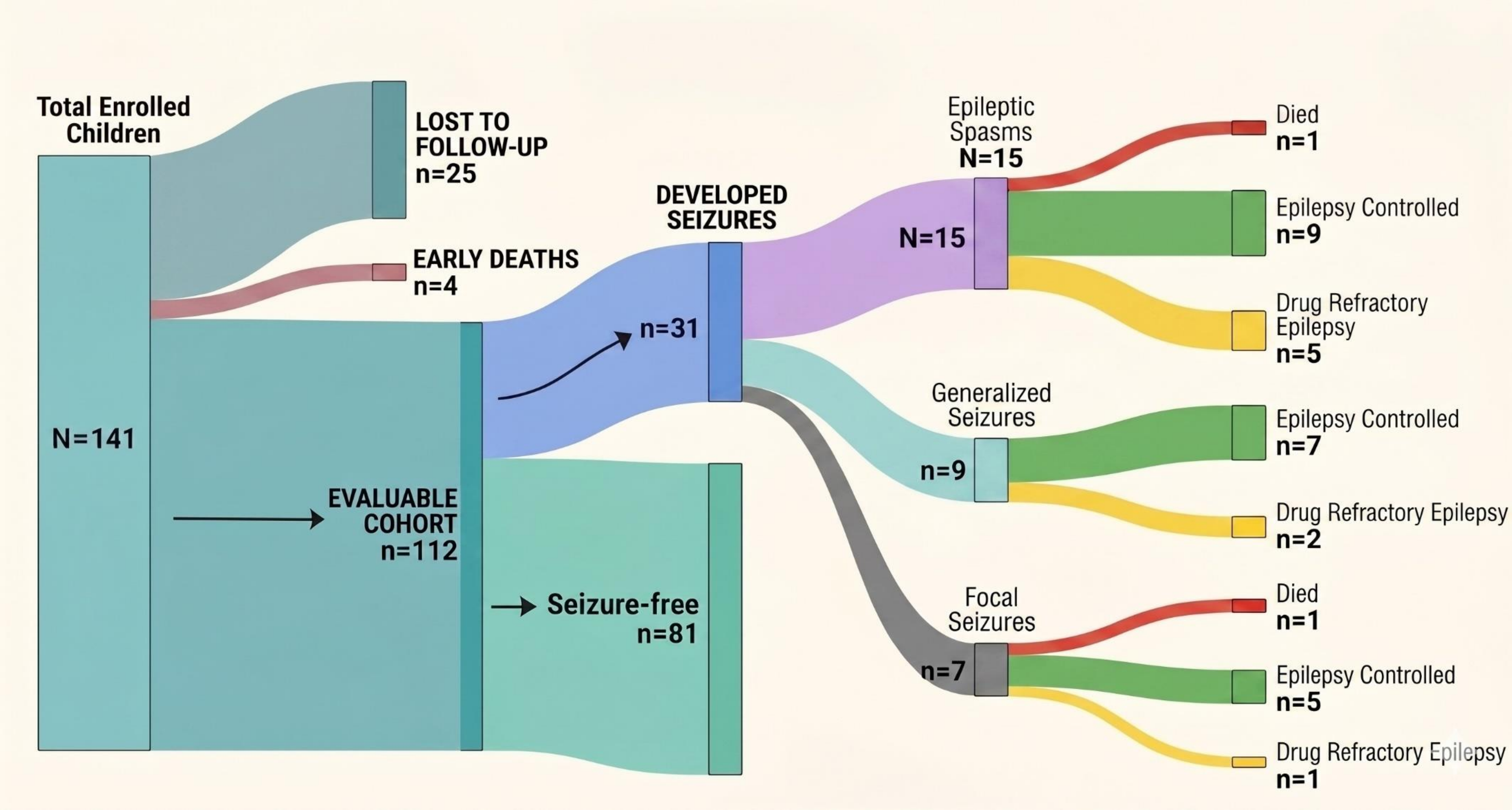

Supplemental Figure 2: Flow diagram of literature search and selection of studies

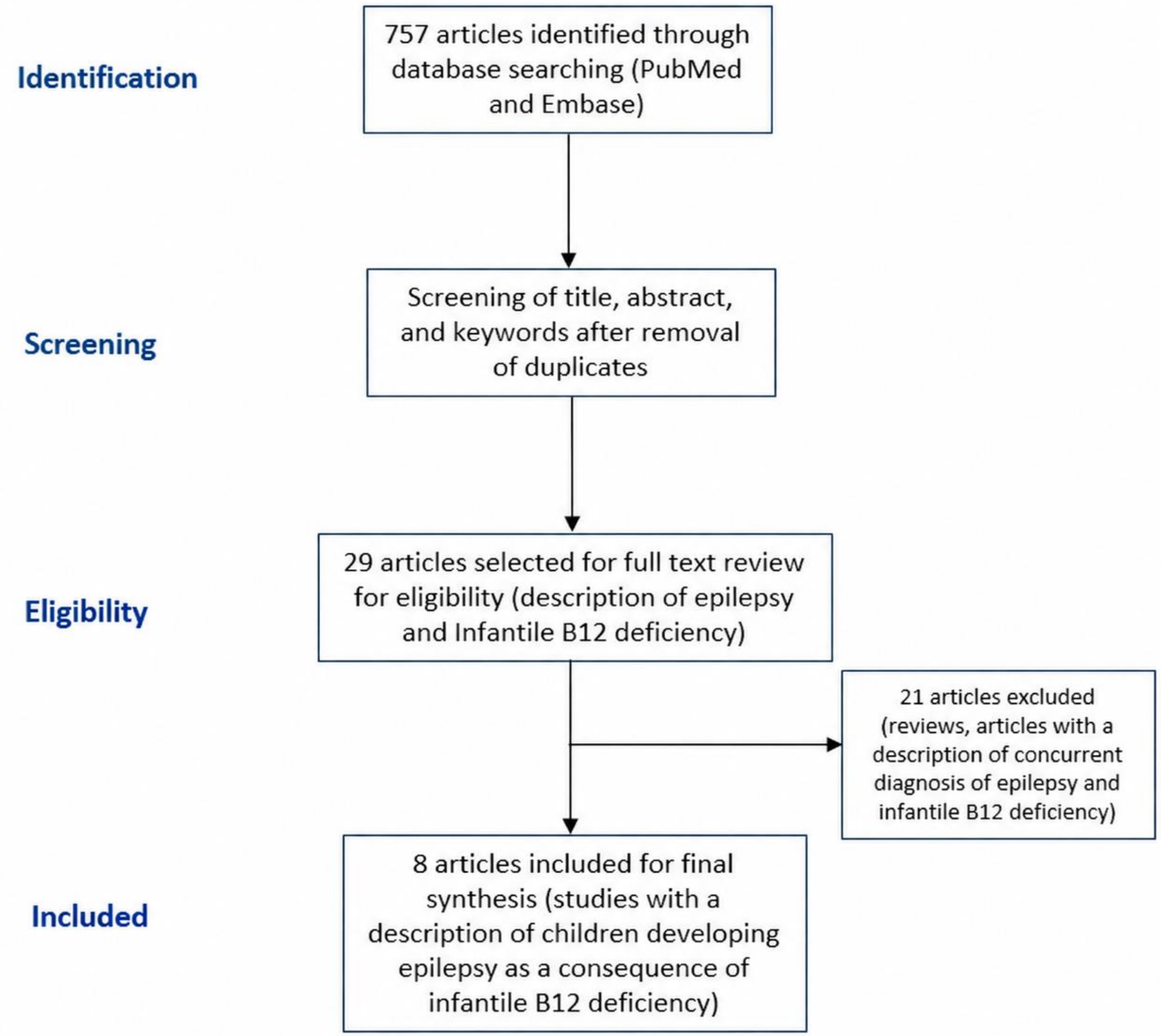

Supplemental Table 1: Summary of published studies on Epilepsy as a long-term sequela among children with infantile Vitamin B12 deficiency

Study & year	Type of study	Number of patients	Age at		Sex	Time interval from diagnosis of B12 deficiency to onset of epilepsy	Type(s) of seizures	EEG	ASM Treatment received & duration	Treatment response	Neurodevelopmental outcome	Incidence of epilepsy
			Diagnosis of B12 deficiency	Onset of epilepsy								
Honzik et al. 2010	Prospective	40	Patient 1: 6.5 m	7 m	NA	0.5 m	Epileptic spasms	NA	NA	Resolved	Mild to moderate Mental Retardation in both	2/40
			Patient 2: 6 m	7.5 m	NA	1.5 m	Epileptic spasms	NA	NA	Resolved	Mild to moderate Mental Retardation in both	
Glaser et al. 2015	Case report	1	6 m	7.5 m	F	1.5 m	Epileptic spasms	Hypsarrhythmia	B6 (failed), Sultiam	Electroclinical resolution after 6 weeks of Sultiam	Seizure-free, IQ – 91 (normal) at 8 years of age	-
Fadilah et al. 2018	Case series	7	5 m	8 m	NA	3 m	Epileptic spasms	NA	Prednisolone	Responded	Speech and language impairment (resolved by 2-3 years)	1/7
Azad et al. 2020	Case series	5	6 m	15 m	NA	12 m	Epileptic spasms	Hypsarrhythmia	ACTH	Complete resolution	NA	-
			10 m	12 m	NA	2 m	Epileptic spasms	Hypsarrhythmia	ACTH	Complete resolution	NA	
			9 m	15 m	NA	6 m	Epileptic spasms	Hypsarrhythmia	ACTH	Complete resolution	NA	
			8 m	18 m	NA	10 m	Epileptic spasms	Hypsarrhythmia	ACTH, VGB, VPA	Partial response	NA	
			10 m	15 m	NA	5 m	Epileptic spasms	Hypsarrhythmia	ACTH, VGB	Complete resolution	NA	

Pavone et al. 2021	Case series	3	7 m	8.5 m	M	1.5 m	Epileptic spasms	Hypsarrhythmia	TPM	Clinical resolution, EEG – bilateral frontal spikes and waves	NA	1/3
Goraya et al. 2023	Case series	3	13 m	16 m	F	3 m	Epileptic spasms, Focal seizures with impaired awareness	Hypsarrhythmia	Prednisolone, VPA, TPM	Partial response to prednisolone, complete resolution after VPA and TPM	Seizure-free at 42 months of age, and language & cognitive deficits	-
			6 m	10 m	M	4 m	Epileptic spasms	Hypsarrhythmia	Prednisolone (withdrawn prematurely), CZP, VPA, LEV	Complete resolution after 4 months	Seizure-free at 3 years of age. Language delay and autistic features	
			7 m	11 m	F	4 m	Epileptic spasms	Hypsarrhythmia	Prednisolone	Complete resolution in 3 days	Spasm-free at 1-month follow-up	
Sharawat et al. 2023	Retrospective cohort	21	NA	Median age: 6.5 m (IQR: 5-8 m)	NA	NA	Epileptic spasms	Hypsarrhythmia – 7 patients, Hypsarrhythmia variant – 8, focal/multifocal epileptiform abnormality – 6	Hormonal therapy – 7, other ASMs – 4	After 28 d, Complete spasm freedom – 18, Incomplete (>50% reduction – 3, <50% reduction – 0)	All remained spasms and seizure-free at 6-month follow-up. Baseline DQ – 47 (IQR: 38-63)<br><br>Follow-up DQ after 6 m – 66 (IQR: 53-89) Change in DQ from baseline to follow-up – 18 (IQR: 14-25)	-
Juhi et al. 2025	Cross-sectional study	35	7 m	8 m	NA	1 m	Epileptic spasms	NA	ACTH	Complete remission	NA	1/35
Present study 2026	Prospective cohort	141	Median-13 mo	4.7 ± 3.2 mo after treatment		4.7 ± 3.2 mo	Epileptic spasms; generalized seizures, focal seizures	As per the seizure type		Spasms resolved in all, overall epilepsy in remission in 21(68%)	Lower DQ than those without epilepsy	28%

Supplemental Table 2: Comparing features at diagnosis and follow-up in children with epilepsy and those without epilepsy who were diagnosed with infantile vitamin B12 deficiency

**Parameter**	**Epilepsy (n=29)**	**No epilepsy (n= 81)**	**P value**
Age at diagnosis (months)	14.9 ± 4.5	13.3 ± 4.8	0.121
Boys	17	42	0.665
Developmental delay	28	74	0.678
Milestone regression	19	44	0.383
Pallor	29	79	1.000
Lethargy	27	74	1.000
Skin Hyperpigmentation	26	73	1.000
Tremors	17	40	0.516
Seizures	5	0	**<0.001**
Head circumference for age (Z-score)	-1.94 ± 1.16	-1.96 ± 1.36	0.961
Hemoglobin (g/dL)<br>Plasma homocysteine levels(μmol/L)	8.3 ± 1.8<br>44.1 ± 22.3	7.7 ± 2.2<br>61.25 ± 27.7	0.199<br>0.004
**MRI at diagnosis**			
Frontotemporal atrophy	27	73	1.000
White matter atrophy	23	69	0.559
Corpus Callosum Atrophy	24	72	0.516
Basal Ganglia Signal changes	5	3	**0.029**
Cerebellar atrophy	22	70	0.242
**MRI at follow-up**			
Frontotemporal atrophy	9	19	0.451
White matter atrophy	4	15	0.776

Corpus Callosum Atrophy	12	26	0.373
Cerebellar atrophy	5	16	1.000
**Developmental Assessment**			
Baseline full-scale DQ	20.4 ± 12.6	24.5 ± 10.8	0.091
Follow up full-scale DQ	44.3 ± 8.8	48.7 ± 8.6	**0.027**

*The six children who died have been excluded from this analysis

Supplemental table 3: Patient-wise treatment response and follow-up in those children who developed Infantile Epileptic Spasms Syndrome (IESS)

No.	Child code	ACTH response/timing	Vigabatrin use	Time to complete clinical response	Relapse of epileptic spasms	Later seizure outcome / Status at last follow-up
1	Y	Partial response during the first 2 weeks	Added on Day 14 of ACTH; continued for 3 months	Complete response after 10 days of VGB	No relapse	Later generalized seizures; subsequently died
2	R	Complete response	Not required	7-10 days of ACTH	No relapse	No later non-spasm seizures reported; seizure-free at last follow-up
3	A	Partial response	Added on Day 10 of ACTH; continued for 4 months, then tapered and stopped	Complete response after 7-10 days of VGB	No relapse	No later seizures reported
4	J	Complete response	Not required	Within 14 days of ACTH	No relapse	Later generalized seizures; seizure-free at last follow-up
5	J2	Complete response	Not required	12 days of ACTH	No relapse	Currently seizure-free
6	S	Partial response	Added on Day 14 of ACTH; continued for approximately 4 months, then tapered and stopped	Complete response after 10 days of VGB	No relapse	No later seizures reported
7	A2	Complete response	Not required	10 days of ACTH	No relapse	No later seizures reported
8	S2	Complete response	Not required	8 days of ACTH	No relapse	No later seizures reported
9	K	Complete response	Not required	7-10 days of ACTH	No relapse	No later seizures reported
10	H	Complete response	Not required	10 days of ACTH	No relapse	No later seizures reported
11	V	Complete response	Not required	7 days of ACTH	No relapse	No later seizures reported
12	D	Partial response by Day 12	Added on Day 12 of ACTH; continued for 4–5 months, then tapered and stopped	Exact time to complete response after VGB: to be entered	No relapse	Later generalized seizures; seizure-free at last follow-up
13	A3	Complete response	Not required	7 days of ACTH	No relapse	No later seizures reported
14	Z	Complete response	Not required	10 days of ACTH	No relapse	Generalized seizures plus febrile seizures; seizure-free at last follow-up
15	M	Complete response	Not required	10 days of ACTH	No relapse	Generalized seizures plus febrile seizures; seizure-free at last follow-up
